# Recent Advances in Macrocyclic Fluorescent Probes for Ion Sensing

**DOI:** 10.3390/molecules22020200

**Published:** 2017-01-25

**Authors:** Joseph K.-H. Wong, Matthew H. Todd, Peter J. Rutledge

**Affiliations:** School of Chemistry, The University of Sydney, Sydney, New South Wales 2006, Australia; joseph.wong@sydney.edu.au (J.K.-H.W.); matthew.todd@sydney.edu.au (M.H.T.)

**Keywords:** chemosensor, fluorescence sensing, fluoroionophore, spectroscopy, molecular probes

## Abstract

Small-molecule fluorescent probes play a myriad of important roles in chemical sensing. Many such systems incorporating a receptor component designed to recognise and bind a specific analyte, and a reporter or transducer component which signals the binding event with a change in fluorescence output have been developed. Fluorescent probes use a variety of mechanisms to transmit the binding event to the reporter unit, including photoinduced electron transfer (PET), charge transfer (CT), Förster resonance energy transfer (FRET), excimer formation, and aggregation induced emission (AIE) or aggregation caused quenching (ACQ). These systems respond to a wide array of potential analytes including protons, metal cations, anions, carbohydrates, and other biomolecules. This review surveys important new fluorescence-based probes for these and other analytes that have been reported over the past five years, focusing on the most widely exploited macrocyclic recognition components, those based on cyclam, calixarenes, cyclodextrins and crown ethers; other macrocyclic and non-macrocyclic receptors are also discussed.

## 1. Introduction

**Figure 1:**
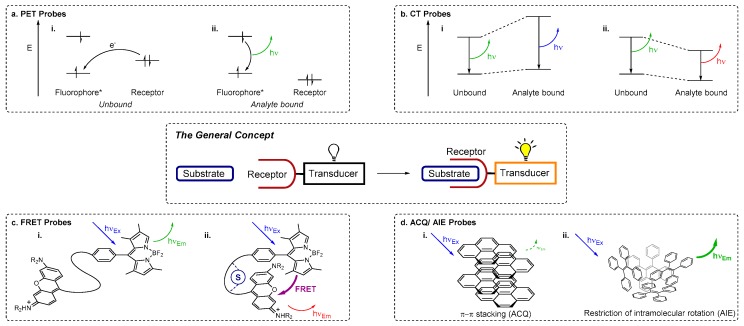
Schematic representation of a receptor-substrate interaction transduced into a detectable signal (central panel) and the key concepts underpinning important mechanisms of fluorescence sensing. (**a**) *Photoinduced Electron Transfer (PET)*: frontier orbital energy diagrams showing how (i) PET from the receptor quenches the fluorescence output of the fluorophore (the transducer); but (ii) when the substrate/analyte binds to the receptor, PET is inhibited and fluorescence emission turned on; (**b**) *Charge Transfer (CT)*: frontier orbital diagrams representing the energy levels of CT systems and changes in emission wavelength due to (i) a reduced dipole moment (blue-shifted fluorescence emission) and (ii) an enhanced dipole moment (red-shifted fluorescence emission); (**c**) *Förster Resonance Energy Transfer (FRET)*: (i) two π systems that are too far apart for FRET to occur in the absence of substrate; are (ii) brought into proximity when the substrate (S) binds, enabling the FRET process and triggering a change in the emission wavelength; (**d**) *Aggregation Caused Quenching (ACQ) and Aggregation Induced Emission (AIE)*: schematic representations of (i) ACQ resulting from the stacking of pyrene units and (ii) AIE that occurs when the ‘rotors’ of hexaphenylsilole (HPS) interlock. See text for further discussion of these sensing mechanisms.

A chemical sensor provides analytical data about species present in a chemical system and consists of two essential components—a receptor, which binds the substrate/analyte, and a transducer, which reports this binding event [1]. For a chemosensor, defined as *a molecule of abiotic origin that signals the presence of matter or energy* [2], the receptor interacts with the species of interest to trigger a detectable signal from the transducer, which reports useful information (Figure 1, central panel) [3,4]. Molecular chemosensors offer the key advantage that they may be constructed using the tools of synthetic chemistry, and thus readily modified to alter either the selectivity of the receptor or the sensitivity and output of the transducer. A diverse range of chemosensor probes has been developed based on a variety of structures ranging from small molecules, metal complexes and macrocycles through to polymers, carbon nanotubes, quantum dots and nanoparticles. These systems have been developed for the sensing and detection of substrates of varying size and charge, ranging from cations and anions, to small molecules such as explosives, and biologically important motifs [5,6,7,8,9,10,11,12,13,14].

Upon substrate-receptor binding, the interaction is most commonly transduced as an optical or electrochemical signal [7,8,15,16]. An optical change may manifest as either a change to the absorbance profile of the probe, allowing colorimetric determination using UV/Vis spectroscopy, or an enhancement or quenching of the probe’s emission profile, enabling measurement of emission wavelength and intensity by fluorescence spectroscopy. An electrochemical change resulting from a change in current or redox potential may be measured by voltammetry.

The detection of chemical species has great importance in a variety of fields including environmental, medicinal and biological contexts, and security. For example, it is well established that mercury is a highly toxic heavy metal to humans and other organisms, and thus to ensure drinking water does not exceed the World Health Organization’s guideline value of 6 µg/L of inorganic mercury, continuous monitoring of water quality is required to maintain public health and safety [17,18]. Copper species have been implicated in a variety of neurodegenerative diseases including Parkinson’s and Alzheimer’s diseases via the generation of hydrogen peroxide and other reactive oxygen species, and thus sensors that allow biological imaging of copper in specific intracellular components or processes allow further insight to be gained for medical research into related diseases and disorders [19,20,21,22]. Conversely, as an essential trace element, the monitoring of copper in biological systems is important as copper deficiency leads to a variety of diseases in humans including anaemia and leukopenia [23]. From a security standpoint, the proliferation of explosive devices and the risk of terrorist organisations or rogue states obtaining chemical warfare agents mean that probes which can detect such residues quickly, accurately and safely are of greatly increased value [24,25,26,27].

Numerous classes of fluorescent probes have been developed for the detection of a wide variety of analytes via a variety of emission mechanisms. Many excellent reviews have been published in the area, focusing on particular classes of analyte [28,29,30,31,32,33], receptor or transducer architecture [12,34,35,36,37,38,39,40,41], and other aspects of fluorescence sensing [5,42,43,44,45]. This review will focus on selected interesting structural classes of fluorescence-based chemosensors for a variety of analytes reported over the past five years.

## 2. Fluorescence Sensing Mechanisms

Fluorescence spectroscopy may be used for sensing if a fluorophore is employed as the transducer (reporter) component of a chemosensor. Fluorescence methods offer high sensitivity and fast response times and are relatively inexpensive compared to other analytical techniques such as inductively coupled plasma mass spectrometry (ICP-MS) or ICP atomic emission spectroscopy (ICP-AES). A variety of photophysical mechanisms for fluorescence sensing of analytes are known (Figure 1) [44,46], including photoinduced electron transfer (PET), charge transfer (CT), Förster resonance energy transfer (FRET), excimer formation, and the more recently developed aggregation induced emission (AIE) or aggregation caused quenching (ACQ).

PET-based chemosensors consist of a receptor-linker-fluorophore system and operate as ‘off-on’ or intensity-based probes [44,47,48,49]. Upon excitation of the fluorophore, PET occurs from the receptor HOMO to the HOMO of the excited fluorophore (vacated by the irradiation). The previously excited electron is then unable to return to its original ground state; it is instead back-donated to the receptor and fluorescence is quenched (the ‘off’ state) (Figure 1a). When a cation binds, the redox potential of the receptor is raised as electrons are donated from the receptor to the cation. This lowers the energy of the receptor HOMO to below that of the fluorophore HOMO. As a result the PET process is no longer active and the excited electron in the LUMO of the fluorophore can return to its original ground state with fluorescence emission (the ‘on’ state).

Many chemosensors exploit internal (or intramolecular) charge transfer (ICT) pathways [44,48,49], for which fluorescence effects hinge on the combination of electron donating and electron accepting groups within a conjugated π system that incorporates both the fluorophore (transducer) and receptor. Upon excitation, redistribution of electron density (i.e., CT) from the electron donating moiety to the electron acceptor creates a dipole moment within the molecule. When an analyte binds, this dipole may be enhanced or reduced, depending on the nature of the analyte and the electronic relationship between the receptor and the fluorophore. A reduced dipole moment will result in decreased molar absorptivity and blue-shifted absorbance and fluorescence emission (reflecting reduced conjugation of the ICT system which leads to greater destabilisation of the excited state relative to the ground state upon analyte binding). Conversely, an enhanced dipole will result in an increase in molar absorptivity and red-shifted absorbance and fluorescence (on account of enhanced conjugation that stabilises the excited state more than the ground state when the analyte is bound) (Figure 1b). Other charge transfer processes include twisted internal charge transfer (TICT) [50,51] and metal-ligand charge transfer (MLCT) [46,52]. CT pathways are highly dependent on solvent polarity as the arrangement of solvent molecules around the dipole can provide added stabilisation. As changes are commonly observed to the intensity of more than one emission band, CT sensors enable *ratiometric* detection of analytes (whereby changes in the *ratio* of fluorescence emission at different wavelengths—i.e., changes in emission colour—are monitored, meaning the response is independent of probe concentration) [31,49].

Förster resonance energy transfer (FRET) is a non-radiative transfer of energy from an excited energy donor fluorophore to an energy acceptor via long range dipole-dipole interactions [44,46,53,54]. When FRET operates, fluorescence emission from the original excited fluorophore is not observed; instead the acceptor is excited. If a suitable fluorophore is chosen, the wavelength of the emitted light is far red-shifted from the original excitation wavelength of the donor (Figure 1c). The effectiveness of a FRET process is determined by the spectral overlap between the emission profile of the donor and the absorption profile of the acceptor, the distance between the donor and acceptor units (which is ideally in the range 10–100 Å), and the orientation of the dipole moments of donor and acceptor. Thus ratiometric probes can be obtained by using analyte binding to disrupt a FRET process, while using FRET to a non-emissive energy acceptor results in probes with an effective ‘on-off’ response to analyte binding.

Excimers (‘excited state dimers’) are dimers of fluorophores and are formed upon excitation with light, when half-filled orbitals of the excited fluorophore interact with another fluorophore in its ground state via π–π stacking [42,44,48]. Exciplexes (‘excited state complexes’) operate on a similar principle, but are heterodimeric or involve more than two species [55,56]. The emission profiles of excimer-forming systems consist of bands corresponding to the fluorophore monomer and a broad excimer emission band which is red-shifted relative to the original fluorophore emission. Either stacking or dissociation of fluorophore excimers may be perturbed by analyte binding, enabling the use of such systems as ratiometric probes.

Fluorophore aggregation commonly leads to a decrease in fluorescence intensity (i.e., ACQ), due to the formation of species with poorer fluorescence properties [57]. The contrasting phenomenon AIE was first reported by Tang et al. in 2001 [58], working with molecules that incorporate ‘rotors’ such as phenyl groups. These groups undergo movement or rotation in dilute solutions or in the non-aggregated form, enabling non-radiative decay pathways for excited electrons of AIE fluorophores [46,57,59,60,61]. When such molecules aggregate, fluorescence enhancement is observed, triggered by the restriction of motion (RIM) or rotation (RIR) which inhibit non-radiative pathways due to steric interactions, enable radiative decay, and turn on fluorescence (Figure 1d).

## 3. Cyclam-Based Sensors

The azamacrocyclic tetramine cyclam is a versatile ligand that can be easily functionalised and exhibits strong binding to a variety of cations as a result of the macrocyclic effect, which arises from combination of enthalpic and entropic factors [62]. A variety of cyclam-based compounds have found uses and applications in sensing and other fields [63,64,65,66,67].

**Figure 2:**
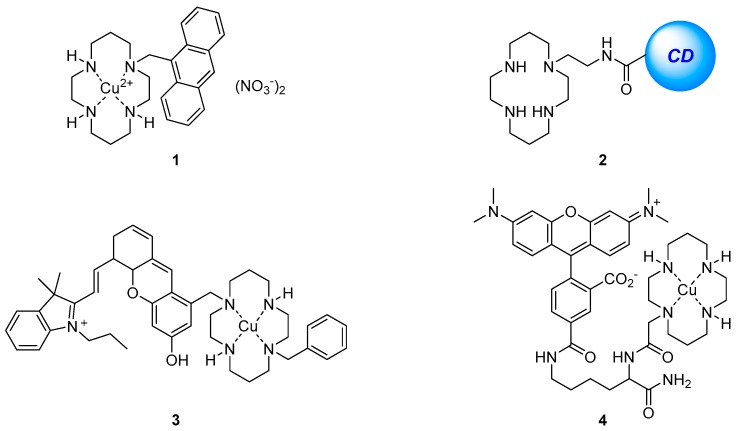
Cyclam-based fluorescent probes for sulfur anions and reactive nitroxyl species. The Cu^2+^ complex **1** functions as a turn-on probe for HS^−^ **1**, with fluorescence enhancement thought to arise due to demetallation of **1** by HS^−^ [68]. The cyclam-functionalised carbon dot system **2** (CD = carbon dot) functions as a probe first for Cu^2+^ (which quenches the intrinsic fluorescence of the CD) and then, using the resultant Cu^2+^ complex, for S^2−^ (which rescues the fluorescence emission, presumably by stripping copper from the macrocycle) [69]. The Cu^2+^-cyclam complexes **3** and **4** function as selective probes for nitroxyl (HNO) for applications in near-infrared fluorescence biological sensing [70,71].

The anthracene-substituted Cu^2+^-cyclam complex **1** has been reported as an ‘off-on’ sensor for HS^−^ (Figure 2) [68]. Screening the fluorescence response upon the addition of a variety of different anions, biothiols and oxidants to the weakly fluorescent **1** revealed that the complex is sensitive to HS^−^ in aqueous media with an observed 5.5-fold fluorescence increase upon the addition of up to 100 equivalents of HS^−^. The resulting emission spectrum after the addition of HS^−^ was nearly identical to that of the parent cyclam ligand, suggesting the fluorescence increase is due to the demetallation of **1** by HS^−^. Fluorescence imaging of HS^−^-spiked HeLa cells pre-incubated with **1** also demonstrated a significant response to increasing HS^−^ concentration. 

The detection of the similar anion S^2−^ through the use of another Cu^2+^-cyclam complex has been developed based on a surface-functionalised carbon dot system **2** [69]. The metal-free sensor **2** displayed good selectivity and sensitivity to the coordination of Cu^2+^ in water with effective fluorescence quenching observed over a variety of competing cations tested, with the exception of Fe^3+^. A screen of the resulting **2**·Cu^2+^ complex with a range of anions revealed that S^2−^ alone led to a fluorescence increase and restoration of the original emission profile of the sensor **2**, thought to occur via demetallation and formation of CuS. This ‘on-off-on’ fluorescence change in response to Cu^2+^ and S^2−^ is attributed to a FRET process which takes place from the carbon dot to the Cu^2+^-cyclam complex. As the Cu^2+^-cyclam complex is non-emissive, the original fluorescence from the carbon dot would be supressed by FRET and consequently regeneration of the free cyclam ligand upon S^2−^ binding revives the carbon dot fluorescence. The utility of this system was demonstrated in the detection of Cu^2+^ in blood serum and tap water, while its low cytotoxicity has been exploited for the successful fluorescence confocal imaging of Cu^2+^ and S^2−^ in spiked HeLa cells.

The Cu^2+^-cyclam complex **3** which bears a near-infrared fluorophore dihydroxanthene (Figure 2) has been reported as a selective sensor for nitroxyl (HNO), the one-electron reduced form of nitric oxide (NO), for applications in near-infrared fluorescence biological sensing [70]. The sensor **3** was shown to be selective for Angeli’s salt, an HNO donor, over a variety of analytes including NO and thiols with a 5-fold fluorescence enhancement of the emission at 715 nm after addition of excess (100 equivalents) Angeli’s salt. Mechanistic studies by cyclic voltammetry, EPR and MS suggested that when **3** binds HNO, the Cu^2+^ is reduced to Cu^+^ leading to demetallation and a consequent increase in fluorescence emission. The near-infrared emission profile of the sensor **3** allowed it to be used in multicolour live cell imaging in conjunction with the green fluorescent sensor ZP1 for the simultaneous sensing of HNO and downstream increases in Zn^2+^ concentration in response to the presence of HNO. The Cu^2+^-cyclam complex **4** has also been used for the sensing of HNO, and reported by the same group [71]. The lysine backbone was assembled on a solid phase resin, coupled with rhodamine and cyclam, cleaved from the resin using TFA and finally complexed with Cu^2+^ to afford **4**. The sensor was selective for HNO and demonstrated a 4-fold fluorescence enhancement of the tetramethylrhodamine emission at 580 nm after the addition of an excess (200 equiv.) of Angeli’s salt. However, in contrast to **3**, mechanistic studies revealed that upon binding and reduction of Cu^2+^ to Cu^+^ by HNO, a fast re-oxidation process occurred with retention of Cu^2+^ in the cyclam. Sensor **4** was also shown to be non-cytotoxic and has been applied to the intracellular sensing of HNO levels in HeLa cells.

**Figure 3:**
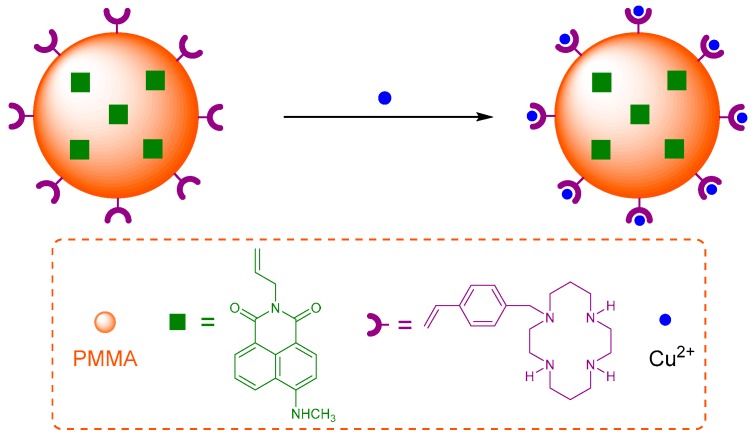
Schematic representation of binding of Cu^2+^ to the polymeric nanoparticle **5**, synthesised in one-pot via mini-emulsion polymerisation. The naphthalimide reporter is covalently bound inside the poly(methyl methacrylate) (PMMA) nanoparticle, and the Cu^2+^ receptor (vinylbenzylcyclam) is covalently attached to the polymer surface [72].

The cation binding affinity of cyclam has been exploited in a polymeric nanoparticle for Cu^2+^ sensing [72]. Sensor **5** was synthesised by a one-pot mini-emulsion polymerisation to include the naphthalimide fluorophore covalently bound inside the poly(methyl methacrylate) (PMMA) nanoparticle, with vinylbenzylcyclam attached to the PMMA surface as the receptor for Cu^2+^ (Figure 3). Sensor **5** was shown to tolerate a reasonably wide pH range (4 to 7) with good photostability after storage for >45 days. It is selective for Cu^2+^ in competition experiments with other cations which are known to form complexes with cyclam in aqueous solution (Zn^2+^, Ni^2+^, Co^2+^, Hg^2+^, Mn^2+^) and produced a decrease in fluorescence output at 505 nm with a 500 nM detection limit. The fluorescence quenching response is due to the establishment of FRET between the donor PMMA and the acceptor Cu^2+^-cyclam which is not emissive.

**Figure 4:**
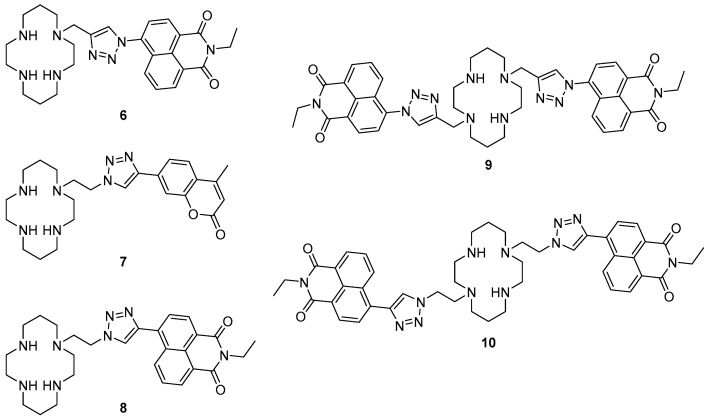
Cyclam-based metal ion probes that incorporate a ‘click’-derived triazole as both a linker between receptor and reporter, and an additional metal-binding ligand (i.e., part of the receptor). The simple naphthalimide systems **6** and **8** respond to Zn^2+^ while coumarin **7** is a Cu^2+^/Hg^2+^ probe. The bis-naphthalimides **9** and **10** are also ‘off-on’ probes for Zn^2+^, but their fluorescence properties display markedly different solvent-dependence. Note the difference in triazole connectivity between **6** and **9** in which the naphthalimide is attached to triazole *N*1, versus **7**, **8** and **10**, in which the fluorophore is linked to *C*4 of the triazole.

A number of cyclam-based chemosensors have been developed for metal ions using the copper-catalysed azide alkyne cycloaddition (CuAAC) reaction to install triazoles as both a linker (between the cyclam and the fluorophore components) and as part of the metal-binding receptor unit (Figure 4). Tamanini et al. developed the first such system, mono-naphthalimide **6** as a turn-on probe for Zn^2+^ [73,74]. Sensor **6** demonstrated excellent selectivity of Zn^2+^ in competition experiments with other cations in aqueous solvent (pH 7), with a 6-fold enhancement of the emission band at 407 nm upon zinc binding; this probe is effective over a wide range of pH (>4.5). It was postulated that PET from the cyclam/triazole unit to the fluorophore in the free ligand quenches naphthalimide fluorescence, and then Zn^2+^ binding interferes with this PET process and the fluorescence output is enhanced. Moderate fluorescence quenching was also observed in response to Cu^2+^ or Hg^2+^. The utility of this fluorescent sensor has been demonstrated in the detection of increasing free Zn^2+^ concentrations in apoptotic thymocytes.

Replacing the naphthalimide with a coumarin fluorophore enabled detection of and differentiation between Cu^2+^ and Hg^2+^ with the ‘on-off’ cyclam sensor **7**, reported by Lau et al. [75]. This probe contains the ‘reversed’ triazole connectivity, in which the pendant fluorophore is connected to the triazole *C*4 (rather than *N*1, as in **6**), synthesised by combining the fluorophore-alkyne with an azido-cyclam building block. Sensor **7** was selective for Cu^2+^ and Hg^2+^ in competition experiments with other cations tested, and responded to these ions with quenching of the fluorescence emission at 389 nm. The observed quenching of fluorescence was rationalised as arising from paramagnetic and heavy metal effects. Zn^2+^ also bound to this probe, and when present in significant excess (50 fold) interfered slightly with the quenching response to Cu^2+^ and Hg^2+^. Differentiation between Cu^2+^ and Hg^2+^ was achieved by the addition of S_2_O_3_^2−^ which to led to the revival of the fluorescence for **7**·Hg^2+^ but not **7**·Cu^2+^. ^1^H-NMR and MS experiments revealed the demetallation of the **7**·Hg^2+^ complex under these conditions, which reactivates the fluorescence output of **7**.

Further work by Ast et al. demonstrated that the triazole connectivity has a marked influence on the fluorescence properties of this class of probes [76,77]. Mononaphthalimide derivative **8** has the ‘reversed’ *C*4 connectivity to the fluorophore and was found to respond strongly to zinc, like the analogous *N*1-linked probe **6**, showing a 5-fold enhancement of the fluorescence emission at 458 nm in response to Zn^2+^ in aqueous solvent (pH 7.4). However, fluorescence quantum yield and lifetime measurements revealed that the change in triazole connectivity gave rise to a quantum yield 10 times higher and a fluorescence lifetime 6 times longer for probe **8** than probe **6** (which has the ‘original’ triazole connectivity). Differences in the solvatochromaticity of probes **6** and **8** (in particular, differences in the solvent dependence of their emission wavelengths) indicated that while PET suppression upon Zn^2+^ binding underpins the turn-on response of **6**, the response of probe **8** to Zn^2+^ arises from interruption of a TICT process by Zn^2+^ coordination [76]. Additional fluorescence experiments were undertaken at low temperatures with the Zn^2+^ and Cu^2+^ complexes of the mono-naphthalimide and coumarin sensors, demonstrating that PET is predominately responsible for the fluorescence response to Zn^2+^ while quenching due to Cu^2+^ is due to energy transfer [77].

When a second triazolylnaphthalimide unit was built into the structure, the symmetrical bis-naphthalimide **9** was as selective as the original probe **6** for Zn^2+^ over a variety of cations tested in 7:3 water/MeCN buffered with HEPES at pH 7; moderate quenching was again observed in response to Cu^2+^ or Hg^2+^. The inclusion of a second triazolylnaphthalimide unit in **9** lent this probe twice the fluorescence output of the mononaphthalimide **6**: a 12.7-fold fluorescence increase of the emission at 416 nm was observed in response to Zn^2+^ binding, providing acetonitrile was present in the analyte solution. Wong et al. recently described the synthesis and characterisation of a second bis-naphthalimide **10**, the reversed triazole analogue of **9** [78]. In contrast to the previously reported naphthalimide sensors **6**, **8** and **9**, probe **10** demonstrated poor response to Zn^2+^ in aqueous solvent (pH 7.4). However, a 22-fold fluorescence enhancement at 420 nm in response to Zn^2+^ was observed in acetonitrile. Investigation of the photophysical behaviour of bis- and mononaphthalimide systems revealed that both the reversal of triazole connectivity (naphthalimide connected via triazole *C*4 vs. *N*1) and incorporation of the second triazolylnaphthalimide unit influence the properties of these probes. With the aid of single crystal X-ray structures, it was postulated that the contrasting photophysical behaviour of these bisnaphthalimide systems arises due to differences in the coordination pattern of the pendant triazoles to Zn^2+^: while both triazoles coordinate to the metal in the zinc complex of ligand **10**, only one of the pendants is bound to zinc in the complex formed by **9**.

**Figure 5:**
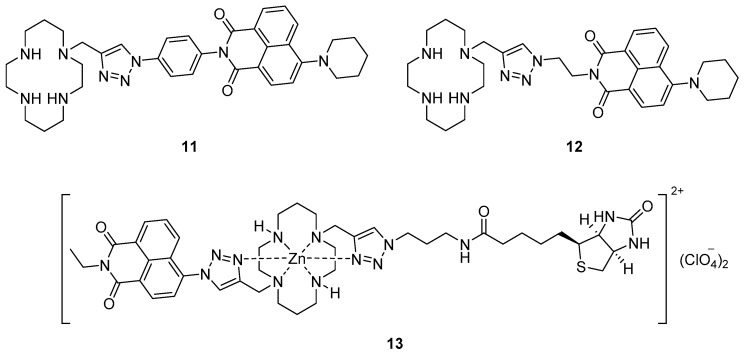
The cyclam-piperidinylnaphthalimide conjugates **11** and **12** are turn-off probes for Cu^2+^, while the Zn^2+^ complex **13** of a biotinylated cyclam-naphthalimide enables visualization of the biotin/ avidin binding interaction using fluorescence.

Yu et al. reported the incorporation of a piperidinyl group at the 4-position of 1,8-naphthalimides in sensors such as **11** and **12** (Figure 5) [79]. These and related probes with reversed triazole connectivity were selective for Cu^2+^ with significant quenching of the fluorescence emissions around 545–558 nm in aqueous solvent (pH 7.4). Surveying the response of these probes in a range of solvents revealed a high degree of solvatochromaticity, with significant blue-shifts in emission maxima (>50 nm difference) and smaller Stokes shifts (>1300 cm^−1^ difference) when switching from polar (e.g., water) to non-polar (e.g., toluene) solvents. The potential of these systems for application to biological imaging was investigated with no significant cytotoxicity observed. Attempts to include *F*-BODIPY fluorophores in systems of this type have hitherto proved unsuccessful, with the conditions required to deprotect the cyclam nitrogen atoms also stripping boron from the fluorophore in the final step of the synthesis [80].

Extending this approach one step further, Yu et al. used the Zn^2+^ complex **13** of a biotinylated cyclam-naphthalimide ligand to characterise the biotin/avidin binding interaction using fluorescence, i.e., to create a fluorescent probe that responds to the protein avidin [81]. When the complex **13** was introduced to a buffered avidin solution, significant quenching of the fluorescence emission was observed relative to control solutions, but only up to a 4:1 ratio of **13**:avidin. This quenching is quantitatively associated with the biotin-avidin binding event, which occurs with a 4:1 stoichiometry [82]. It was proposed that the fluoresence response of **13** to avidin arises from changes in Zn^2+^-coordination by the biotinylated triazole upon protein binding, which affects the fluorescence mechanisms in operation.

## 4. Calixarene-Based Sensors

**Figure 6:**
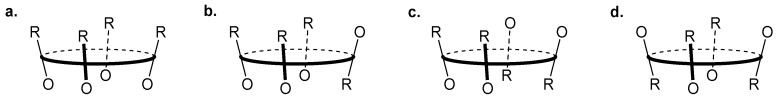
Schematic representation of the four conformations of calix[4]arene which show the different possible orientations of the phenolic oxygens: (**a**) cone; (**b**) partial cone; (**c**) 1,2-alternate; (**d**) 1,3-alternate [83].

Calixarenes are cyclic phenol oligomers that are characterised by a structurally rigid 3-dimensional scaffold containing an interior cavity; they may adopt different conformations depending on size (the number of repeating units in the macrocycle) and substituents on the upper and lower (OH side) rims of the chalice-like structure. For example calix[4]arenes are comprised of four phenol monomers and can exist as cone, partial-cone, 1,2-alternate or 1,3-alternate conformations (Figure 6); for simple calix[4]arenes the cone conformation is preferred at room temperature, but conformationally mobile [83]. Thus calixarenes may be functionalised to generate molecular probes with various orientations of receptor and/or reporter motifs, and fluorescent chemosensors based on a calixarene core have been developed as robust receptors for a range of different analytes [84,85,86].

**Figure 7:**
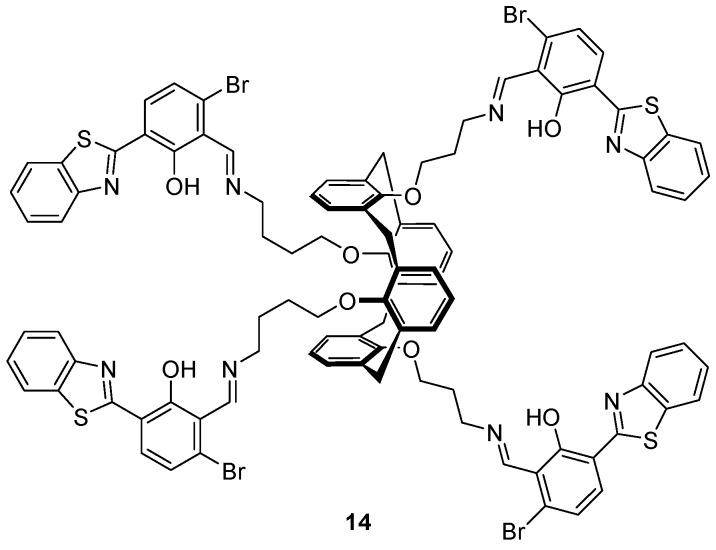
The benzothiazole-substituted calix[4]arene probe **14** presents the 1,3-alternate conformation. This probe functions as a turn-off sensor for Cu^2+^, binding two equivalents of this metal ion which quench its fluorescence output. The resulting copper complex is an effective turn-on sensor for S^2−^, which—alone among anions tested—rescues the fluorescence of **14**. Alternatively, **14** also functions as a turn-on probe for HSO_4_^−^ which hydrolyses the imines and thereby enhances the fluorescence output [87].

The benzothiazole-substituted calix[4]arene **14** bearing a 1,3-alternate conformation (Figure 7) was investigated as a sensor for Cu^2+^, S^2−^ and HSO_4_^−^ by Erdemir et al. [87], and found to be an ‘on-off-on’ fluorescence sensor for Cu^2+^ and S^2−^. Selective binding of Cu^2+^ was achieved over an assortment of cations tested and led to the formation of a 1:2 **14**:Cu^2+^ complex with a 90-fold decrease in the 542 nm fluorescence emission band upon the addition of 20 equivalents of Cu^2+^. Addition of a variety of anions to the **14**·Cu^2+^ complex revealed that only S^2−^ led to the revival of fluorescence to the initial level of **14**. Additionally, **14** was shown to be an ‘off-on’ sensor for HSO_4_^−^ over a variety of anions (including OH^−^, vide infra) in competitive binding experiments with a 10-fold fluorescence enhancement after the addition of up to 50 equivalents of HSO_4_^−^. ^1^H-NMR and FTIR experiments revealed that after the addition of HSO_4_^−^, the imine is hydrolysed and the benzothiazole fluorophore cleaved off to generate the benzothiazole aldehyde, which is highly emissive.

**Figure 8:**
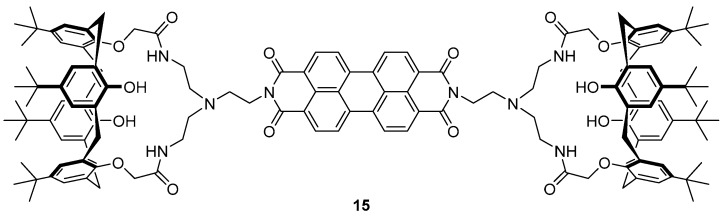
This perylene-based calix[4]arene **15** adopts a cone conformation and functions as a turn-on probe for Hg^2+^. The metal ion coordinates to the three aza-crown nitrogen atoms and the nitrogen of the perylene, thus disrupting PET and enhancing fluorescence output [88].

The same group have reported a calix-aza-crown based sensor **15** for the detection of Hg^2+^ in biological imaging, in which a perylene fluorophore is appended to two calix[4]arene moieties via an aza-crown bridge (Figure 8) [88]. The sensor **15** demonstrated high selectivity towards Hg^2+^ over a variety of different cations tested in competition experiments and fluorescence titrations revealed a 14-fold fluorescence enhancement of emission at 536 and 576 nm upon the addition of 100 equivalents of Hg^2+^, via the formation of a 2:1 Hg^2+^:**15** complex. Each Hg^2+^ ion was complexed to **15** via three aza-crown nitrogens and a perylene nitrogen, and this binding disrupts the PET process from the aza-crown nitrogens to the perylene fluorophore, leading to a fluorescence increase. The sensor **15** was successfully used to image Hg^2+^ in spiked SW-620 cells.

**Figure 9:**
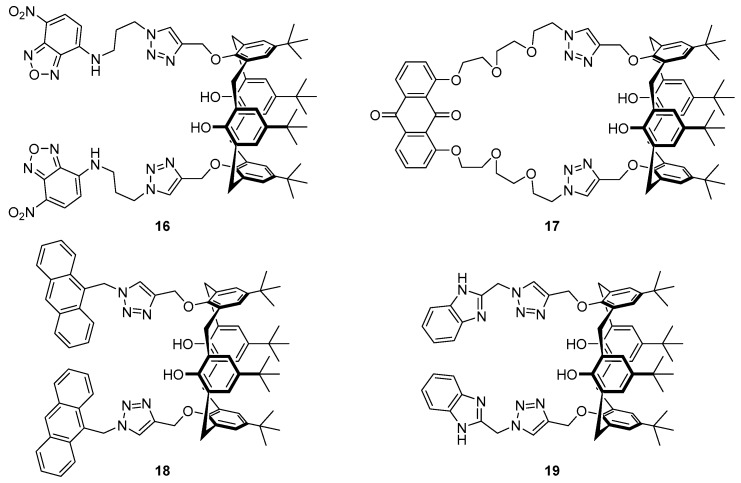
Benzoxadizole-functionalised calix[4]arene probe **16** responds selectively to Ag^+^, which triggers quenching of the free ligand emission at 527 nm and emergence of a new emission band at 576 nm; the resulting metal complex is then an effective probe for HCHO, which reduces the Ag^+^ and regenerates the free probe **16** [89]. The anthraquinone-functionalised calix[4]arene **17** responds selectively to Ca^2+^ with enhanced fluorescence emission at 510 nm, and the resulting **17**·Ca^2+^ complex then functions as a turn-off probe for fluoride [90]. Triazolyl-anthracene derivative **18** is a turn-off probe for Co^2+^ [91], while the structurally related benzimidazole probe **19** binds Cu^2+^ and reports this with a ratiometric fluorescence response (via changes in emissions at 311 and 380 nm) [92].

The incorporation of a 1,2,3-triazole as both the linker and part of the receptor in the *bis*-nitrobenzoxadiazole (NBD) substituted calix[4]arene structure **16** (Figure 9) has been reported to afford a sensor for Ag^+^ and formaldehyde (HCHO) [89]. Alkylation of the precursor calixarene phenols with propargyl bromide followed by CuAAC to attach the NBD azide afforded sensor **16**, which was selective for Ag^+^ over a variety of cations tested. Upon binding with Ag^+^, a fluorescence decrease of the 527 nm emission was observed, along with the emergence of a new emission band at 576 nm with a 14-fold fluorescence increase at this wavelength. Furthermore, the silver complex **16**·Ag^+^ was able to act as a sensor for HCHO via reduction of the Ag^+^ to regenerate the original emission profile of free ligand **16**, with no loss of sensitivity over five iterations of the Ag^+^/HCHO detection cycle. Titration experiments followed by ^1^H-NMR spectroscopy suggested that binding of Ag^+^ to sensor **16** occurs via the *N*3 nitrogens of the two triazoles and this consequently brings together the triazole-NBD fluorophore chains, resulting in the new excimer emission band at 576 nm.

The anthraquinone/calix[4]arene derivative **17** incorporating a similar bis-triazole moiety has been reported by Zhan et al. [90]. Selective binding of sensor **17** to Ca^2+^ was achieved over a variety of other cations and yielded a significant increase in the fluorescence emission at 510 nm. Job’s plot, ^1^H-NMR and MS experiments confirmed the formation of a 1:1 complex, with Ca^2+^ coordinating to the triazole nitrogens, an interaction facilitated by the flexible ether linkers. This coordination results in disruption of PET from the triazole to the anthraquinone fluorophore, leading to the observed fluorescence increase after Ca^2+^ binding. Furthermore, the **17**·Ca^2+^ complex was found to be a selective sensor for F^−^ over other halides, with the addition of F^−^ leading to fluorescence quenching and revival of the original emission profile of **17** via displacement of the bound Ca^2+^ to form CaF_2_.

Calix[4]arene probe **18** (Figure 9), functionalised with anthracene fluorophores via triazole linkers, has been reported by Mummidivarapu et al. for sensing Co^2+^ [91]. This probe was found to be selective for Co^2+^ over a variety of cations tested and gave a 5-fold decrease of the 417 nm emission band with a calculated detection limit of 0.92 µM. Complexation of **18** with Co^2+^ formed a 1:1 complex, confirmed by Job’s plot and MS analysis. The binding mode was determined by ^1^H-NMR titration and DFT calculations which revealed that the triazole plays an important role in coordination of the Co^2+^, which binds to the triazole *N*3 nitrogen and the four oxygens of the calix[4]arene.

The Rao group reported benzimidazole-triazole substituted calix[4]arene **19** (Figure 9) which functions as an ‘off-on-off’ probe dependent on the concentration of Cu^2+^ [92]. This system was shown to be selective for Cu^2+^ via the formation of 1:1 and 2:1 Cu^2+^:**19** complexes and displayed no significant change in competition experiments with the other cations tested. DFT calculations suggested that in both complexes, a Cu^2+^ ion is coordinated to the triazole *N*1 nitrogens and benzimidazole nitrogens, while in the 2:1 complex, the second Cu^2+^ binds to the triazole and calix[4]arene oxygens in an analogous manner to that seen in the **18**·Co^2+^ complex. Probe **19** gives a ratiometric fluorescence response to Cu^2+^, with the addition of up to three equivalents leading to quenching of the original emission maximum at 311 nm along with the appearance of an excimer emission band at 380 nm. Further addition of Cu^2+^ leads to formation of the 2:1 complex with quenching of the 380 nm excimer band in addition to further decreases to the 311 nm emission.

**Figure 10:**
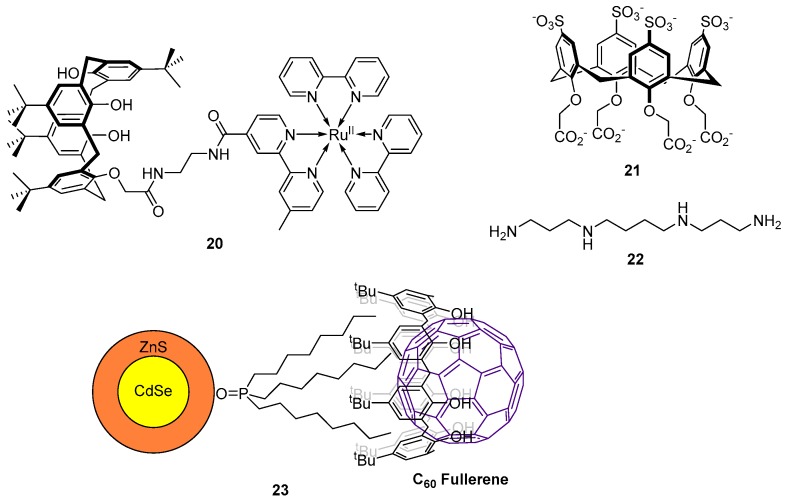
Ruthenium(II)-bipyridine-substituted calix[4]arene sensor **20** is a turn-off probe for CN^−^, which causes fluorescence quenching and a blue-shift of the emission band at 624 nm [93]. The simple, polyanionic calix[4]arene-based sensor **21** is an ‘on-off’ probe for spermine **22** [94], while a QD-appended calix[8]arene receptor forms the complex **23** with C_60_ fullerene, which quenches the QD fluorescence output [95].

More recently, Maity et al. reported a ruthenium(II)-bipyridine-substituted calix[4]arene sensor **20** for the detection of CN^−^ (Figure 10) [93]. Of a range of sodium salts tested, NaCN alone led to fluorescence quenching and a blue-shift of the emission band at 624 nm (in 95:5 H_2_O:MeCN). However, when tetrabutylammonium (TBA) salts of the same anions were tested, TBACN gave a similar fluorescence quenching response, while a fluorescence enhancement was observed upon addition of TBAOAc. The binding was studied using MS and ^1^H-NMR and it was found that two CN^−^ ions or a single AcO^−^ ion coordinate to the amides of the sensor **20** as 2:1 and 1:1 complexes respectively. The differences in selectivity between the Na^+^ and TBA^+^ salts arise due to the steric environment around the lower rim of the calixarene structure and the amides of **20**. The smaller Na^+^ can bind to the lower rim calixarene phenols, thus presenting a sterically challenging environment which hampers coordination of the larger anion AcO^−^ to the amides. Conversely, the larger TBA^+^ cation is not bound to the lower rim and thus coordination of AcO^−^ to the amides can occur. The sensor **20** has been successfully applied in more complex systems with the sensing of CN^−^ in spiked drinking water and saliva, achieved with excellent recovery of the doped analyte.

D’Urso et al. have reported the synthesis of a water soluble octa-anionic calix[4]arene **21** as a sensor for the tetramine spermine **22** (Figure 10) which is responsible for the regulation of cell growth [94]. The binding of **21** with tetra-protonated spermine [**22**·H4]^4+^ was investigated by fluorescence titration which revealed the formation of 2:1 and 1:1 **21**:[**22**·H4]^4+^ complexes and displayed quenching of the fluorescence emission at 310 nm. Further investigation by single crystal X-ray crystallography revealed that the 1:1 binding mode involves the spermine partially hosted inside the cavity of **21** via salt-bridge interactions with the sulfate groups of the upper rim. In contrast, the 2:1 binding mode involves hydrogen bonding and electrostatic attractions with the carboxylate groups on the lower rim.

Using the larger calix[8]arene, Carrillo-Carrión et al. have determined C_60_ fullerene concentrations using fluorescent quantum dots (QDs) [95]. CdSe/ZnS QDs bearing trioctylphosphine oxide chains on the surface were reacted with *p-tert-*butylcalix[8]arene to give a CdSe/ZnS-calix[8]arene coated sensor, with the optimal calix/QD ratio determined to be 12.5:1. Significant quenching of the QD fluorescence is observed when C_60_-fullerene is encapsulated inside the calixarene cavity to form the complex **23** (Figure 10). This probe has been applied to environmental studies with the successful detection of C_60_ from a doped river water sample achieved with excellent percentage recovery of the doped analyte and a low detection limit of 5 µg/L.

## 5. Cyclodextrin Based Sensors

**Figure 11:**
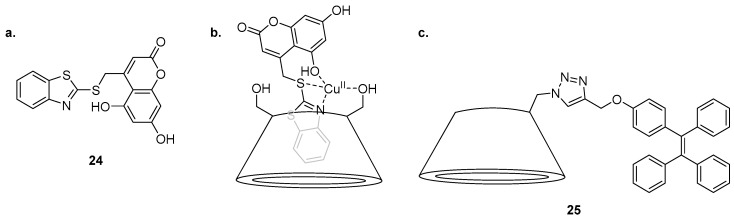
(**a**) The benzothiazole coumarin moiety **24** that combines with β-CD and Cu^2+^ to form an inclusion complex; (**b**) The complex of **24**, β-CD and Cu^2+^ in which the 465 nm emission band of **24** is quenched, thus affording a selective turn-off response to Cu^2+^ [99]; (**c**) Triazole-tetraphenylethylene (TPE) functionalised β-CD **25** is a turn-on probe for Cd^2+^, which causes a significant increase in the fluorescence emission at 476 nm [100].

Cyclodextrins (CDs) are cyclic oligosaccharides composed of α-glucopyranose monomers linked at the 1 and 4 positions, with the three major cyclodextrins bearing 6 (α-CD), 7 (β-CD), or 8 (γ-CD) glucopyranose units [96,97]. The glucopyranose units are arranged to form a cone shape (Figure 11) in which the secondary hydroxyl groups are located on the wider opening and primary hydroxyl groups on the narrower aperture. The interior of a CD is hydrophobic, while the exterior is hydrophilic. As a result CDs are water soluble, but also able to form inclusion complexes with hydrophobic entities inside the cavity; both of these properties are central to their role in molecular probes [98]. With fluorophores covalently linked to the CD or complexed within the cavity, chemosensors with affinity for a variety of analytes can be developed. 

An inclusion complex of a coumarin derivative in β-CD was reported for the sensing of Cu^2+^ by Khan et al. (Figure 11) [99]. Molecular modelling suggested that the structure of the water soluble **24**·β-CD complex contains the benzothiazole group encapsulated inside the β-CD cavity while the coumarin component of **24** protrudes outside. This **24**·β-CD complex is selective for Cu^2+^ over a variety of cations in competition experiments and undergoes quenching of the 465 nm emission band after the addition of Cu^2+^. The **24**·β-CD complex was confirmed to bind to Cu^2+^ as a 1:1 complex by construction of a Job’s plot. Computational studies suggested that the Cu^2+^ ion is coordinated to one primary hydroxyl group of the β-CD, the benzothiazole nitrogen, the sulfur linker and the 5-hydroxyl group of the coumarin. The **24**·β-CD complex was shown to be an effective ‘on-off’ sensor for use in fluorescence confocal microscopy, visualising intracellular Cu^2+^ in doped HeLa cells.

The triazole-tetraphenylethylene (TPE) substituted β-CD **25** has been reported as a highly sensitive sensor for Cd^2+^ exhibiting selectivity for Cd^2+^ in 1:1 water/DMSO solution and returning a significant fluorescence increase at 476 nm over a variety of other competing cations tested [100]. The exception in the competition assay was Ag^+^, which significantly dampened the fluorescence emission in response to Cd^2+^. The sensing mechanism of **25** was proposed to hinge on an aggregation induced emission (AIE) process, a proposal supported by fluorescence emission changes when the water content of the solvent system was varied. As the free ligand **25** is poorly soluble in water, it aggregates and is inherently fluorescent in ≥80% water/DMSO solutions, while the **25**·Cd^2+^ complex afforded no fluorescence output in ≤20% water/DMSO solution; thus the 50% water/DMSO system was used as it provided maximal fluorescence change upon Cd^2+^ binding. Metal binding yields a 2:1 complex, confirmed by a Job’s plot analysis, and was proposed to involve coordination of Cd^2+^ to two molecules of **25**, via the triazole *N*2 nitrogen and a primary hydroxyl group of the β-CD from two different ligand molecules. 

**Figure 12:**
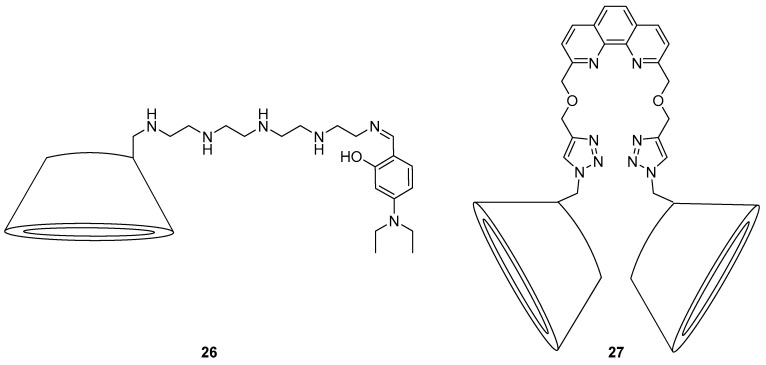
Both of these cyclodextrin-based probes respond selective to Zn^2+^. With **26**, Zn^2+^ is thought to coordinate to the amines and hydroxyl group, triggering fluorescence enhancement and blue shift from 460 nm to 410 nm in water [101]. The *bis*-CD probe **27** responds to Zn^2+^ with fluorescence enhancement and a red-shift in the emission wavelength from 368 to 377 nm [102].

A water soluble β-CD for fluorescence sensing of Zn^2+^ in biological systems was developed by Liu et al. (Figure 12) [101]. Probe **26** was proposed to bind Zn^2+^ selectively through coordination to the 5 amines and the hydroxyl group derived from the diethylamino salicylaldehyde moiety, giving a significant fluorescence enhancement and blue shift from 460 nm to 410 nm in water; the addition of other cations gave no significant fluorescence changes. This sensor was able to penetrate the cell membrane of onion epidermal cells in fluorescence microscopy studies and significant changes to its emission profile as per the in vitro fluorescence studies were evident upon treatment of these cells with Zn^2+^.

A *bis*-β-CD with coordinating triazoles bridged with a phenanthroline fluorophore has been reported as a highly sensitive sensor for Zn^2+^ (Figure 12) [102]. The sensor **27** demonstrated significant fluorescence enhancement and a red-shift from 368 to 377 nm in response to Zn^2+^, with a calculated detection limit of 0.49 µM. Furthermore, the addition of adamantane carboxylic acid (AdCA) resulted in formation of an inclusion complex **27**·AdCA which displayed even greater binding affinity for Zn^2+^ and a lowered limit of detection of 0.34 µM. This increased affinity was proposed to arise from the extra coordination of the carboxylate groups of the **27**·AdCA inclusion complex, in addition to coordination of the phenanthroline nitrogens and the two triazole *N*3 nitrogens to Zn^2+^. Consequently, PET from the triazole nitrogens to the phenanthroline was inhibited, leading to the observed fluorescence changes.

**Figure 13:**
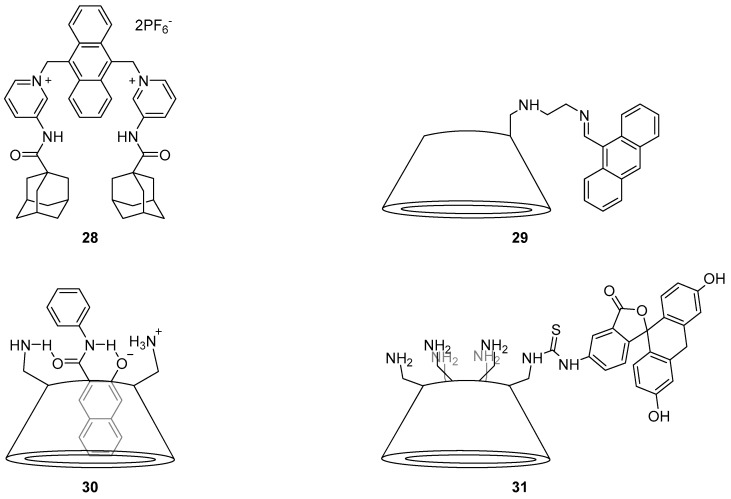
The adamantyl probe **28** gives rise to a strong emission at 500 nm in the presence of H_2_PO_4_^−^, due to formation an exciplex. This is disrupted when β-CD is added and this emission is quenched [103]. The imine-linked anthracenyl system **29** is a turn-on probe for Pb^2+^, thought to operate via a mechanism involving CT from the nitrogen atoms to the fluorophore [104]. Naphthamide β-CD inclusion complex **30** is a selective and highly sensitive probe for Hg^2+^, which triggers a blue shift in fluorescence emission from 577 to 509 nm and quenching of emission intensity, down to a detection limit of 1 pM [105]. The thiourea-linked fluorescein/β-CD derivative **31** has been developed as a probe for 2,4,6-trinitrotoluene (TNT), which quenches the 519 nm emission to a detection limit of 20 nM; TNT is believed to react with the primary amines of **31** to form a Meisenheimer complex which is then involved in FRET with the fluorescein component of the probe [106].

The high affinity of adamantyl groups for β-CD has also been used for sensing H_2_PO_4_^−^ ions, by combining sensor **28** (Figure 13) with β-CD [103]. Probe **28** gave rise to a strong fluorescence signal at 500 nm in response to H_2_PO_4_^−^, showing good selectivity over a variety of other anions (halides, OH^−^, NO_3_^−^ and AcO^−^). This signal is consistent with the general excimer emission of anthracene fluorophores. These fluorescence results and supporting ^1^H-NMR spectroscopic data suggested formation of a **28**·H_2_PO_4_^−^ exciplex via stacking of anthracene fluorophores between **28** units. When β-CD was added to the **28**·H_2_PO_4_^−^ complex, stacking of anthracene units was disrupted, leading to quenching of the exciplex band and a blue shift of the emission wavelength (to 440 nm, which corresponds to the monomer emission of the anthracene fluorophore). No significant fluorescence enhancement was observed when the order of addition was reversed (β-CD, then H_2_PO_4_^−^), suggesting that the **28**·β-CD inclusion complex inhibits assembly of the **28**·H_2_PO_4_^−^ exciplex.

Several heavy metal sensors have been developed using substituted β-CDs. Anthracene-substituted β-CD sensor **29** has been reported to be a sensor for Pb^2+^ by Antony et al. [104]. Fluorescence quenching was observed upon the addition of a large variety of cations to **29**, but a fluorescence enhancement of the 537 nm emission band was observed only after the addition of Pb^2+^. This is likely due to CT from the nitrogen atoms to the anthracene fluorophore. Competitive binding experiments with other quenching cations revealed no significant decrease in the fluorescence response to Pb^2+^, but a small enhancement from Fe^2+^. This suggests that only Pb^2+^ binds strongly to **29**, via coordination to the two nitrogen atoms in the linker to form a 1:1 complex; this was confirmed by a Job’s plot analysis.

Kanagaraj et al. reported the sensing of Hg^2+^ using a naphthamide β-CD inclusion complex **30** (Figure 13) via colorimetric detection and fluorescence [105]. Sensor **30** consists of a 3-hydroxy-*N*-phenyl-2-naphthamide encapsulated inside the hexaamino derivative *per*-6-amino-6-β-CD; this system demonstrated high selectivity for Hg^2+^ over a variety of cations. Binding of Hg^2+^ led to absorption profile changes along with a blue shift in fluorescence emission from 577 to 509 nm and quenching of emission intensity, with a low detection limit of 1 pM. The fluorescence quenching effect was attributed to the disruption of excited-state intramolecular proton transfer (ESIPT). The original fluorescence shown by **30** is due to photoinduced tautomerism of the naphthamide from its enol form ground state to the keto form excited state by proton transfer and subsequent radiative decay to afford fluorescence emission. As Hg^2+^ is coordinated to **30** through the phenoxide oxygen and amide NH of the naphthamide, ESIPT is disrupted and fluorescence quenching occurs. Environmental applications of the sensor **30** were also demonstrated with doped Hg^2+^ in real world water samples.

CD-based chemosensors have also been developed for the detection of explosives in environmental samples. Feng et al. reported the synthesis of a *per*-6-amino-6-β-CD analogue functionalised with fluorescein isothiocyanate (FITC) to form the thiourea-linked fluorescein/β-CD derivative **31**, and evaluated its potential as a probe for 2,4,6-trinitrotoluene (TNT) in water (Figure 13) [106]. The addition of TNT to **31** gives rise to quenching of the 519 nm emission, to a calculated detection limit of 20 nM. The high selectivity of **31** for TNT over a variety of cations found in the environment and the structurally similar dinitrotoluene (DNT) is due to coordination of the amines of **31** to TNT via formation of a Meisenheimer complex. The observed fluorescence changes arise due to FRET with effective overlap of the fluorescein fluorophore’s emission profile with the absorption profile of the **31**·TNT complex, which is non-fluorescent.

## 6. Crown Ether-Based Sensors

Crown ethers are cyclic polyethers which were first reported in 1967 by Pedersen, who subsequently shared the Nobel Prize in 1987 for work in this area [107]. They are oligomers of ethylene glycol which bind strongly to a variety of alkali metal or transition metal cations depending on the size of the macrocyclic ‘crown’ and its central cavity, and have found many applications as sensors, molecular switches and ion chromatographic media [108].

**Figure 14:**
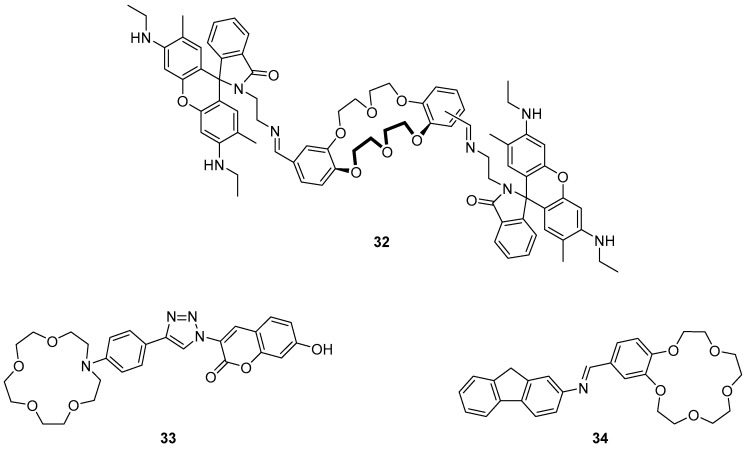
Rhodamine-crown ether conjugate **32** was prepared as a mixture of regioisomers (as indicated), and found to be a selective probe for Cr^3+^; this substrate elicits a turn-on response from the rhodamine fluorophore that is thought to arise via metal-mediated opening of the spirolactam [109]. Triazolyl-coumarin-aza-crown ether **33** responds strongly to Hg^2+^, Fe^3+^ and Cr^3+^, with solvent-dependent variations in response: in water, Fe^3+^ triggers a strong enhancement of the fluorescence emission at 475 nm, with weaker responses to Hg^2+^ and Cr^3+^, while in ethanol it is Hg^2+^ that elicits the strongest response, and smaller responses are seen to Fe^3+^ and Cr^3+^ [110]. Fluorenyl-imine derivative **34** is a very sensitive and selective probe for Cu^2+^, which triggers a ca. 54-fold fluorescence enhancement thought to arise due to inhibition of both PET and C=N isomerisation quenching processes [111].

Diao et al. recently reported a rhodamine-crown ether derivative **32** as a fluorescent sensor for Cr^3+^ (Figure 14) [109]. The high selectivity of this sensor was demonstrated in a screen of environmentally relevant cations, in which only Cr^3+^ produced a strong fluorescence enhancement at 556 nm, while a weak enhancement was observed from Fe^3+^. Additionally, the absorption spectra for the Cr^3+^·**32** mixtures showed an increase of a typical rhodamine absorption band at 532 nm. The observed changes to the absorption and emission profiles result from the formation of a 2:1 Cr^3+^:**32** complex by the coordination of two Cr^3+^ ions to the crown ethers, imine and amide groups, which triggers spirolactam ring opening of the rhodamine. Application of the sensor **32** to the detection of Cr^3+^ in drinking water was demonstrated with spiked Cr^3+^ samples to a detection limit of 1.5 µM. This sensor was extended to fluorescence imaging in biological samples where the sensor **32** was shown to penetrate cell membranes and fluoresce in response to incubation with Cr^3+^.

The coumarin-substituted phenylaza-crown ether **33** (Figure 14), which is constructed around a coordinating triazole by a CuAAC reaction, was reported to be a selective sensor for Hg^2+^, Fe^3+^ and Cr^3+^ [110]. The selectivity of sensor **33** for cations was contingent on the solvent, with a strong fluorescence enhancement at 475 nm in response to Fe^3+^ in water, but a weaker response to Hg^2+^ and Cr^3+^; in contrast in ethanol strong fluorescence enhancement to Hg^2+^ and weaker response to Fe^3+^ and Cr^3+^ was observed. ^1^H-NMR spectroscopic experiments on the **33**·Hg^2+^ complex demonstrated significant downfield movement of the triazole, phenyl and crown ether protons compared with the parent ligand **33**. Molecular energy minimisation calculations revealed that the triazole-coumarin bond is twisted at ca. 30° and this was proposed to act as a ‘virtual’ spacer which enables the PET mechanism. Consequently, coordination of the crown ether and triazole to Hg^2+^ via formation of a 1:1 complex decreases the electron density of the aniline-crown unit and subsequently hinders PET, leading to the observed fluorescence enhancement.

A fluorene-substituted crown-ether **34** has been reported by Chen et al. as a fluorescence ‘off-on’ sensor for Cu^2+^ (Figure 14) [111]. This imine-connected sensor was found to be highly selective for Cu^2+^ in competitive binding experiments with a variety of other cations, and was highly sensitive with a ca. 54-fold fluorescence enhancement upon Cu^2+^ binding and a small blue-shift of the emission from 371 to 380 nm. Fluorescence pH titration, Job’s plot and MS experiments revealed 1:1 binding stoichiometry with Cu^2+^ coordinated in a sandwich complex with the crown ether oxygens and the imine nitrogen of another molecule of **34**. The exceptional fluorescence response of **34** to Cu^2+^ was proposed to occur via the inhibition of both PET and C=N isomerisation quenching processes after complexation.

**Figure 15:**
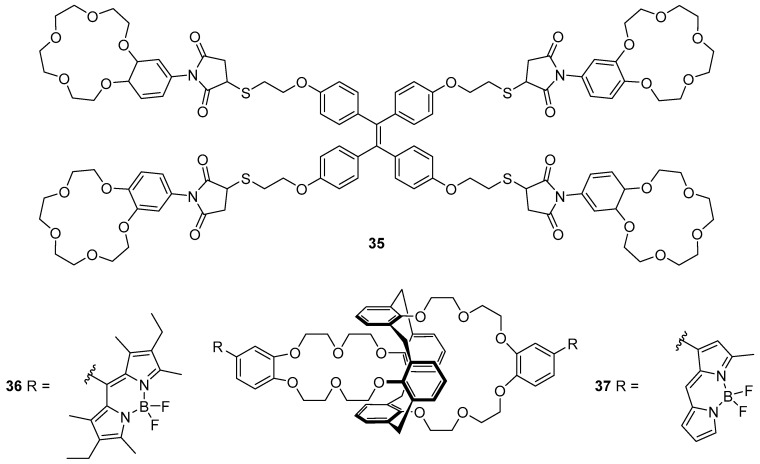
Crown ethers **35**–**37** have proved effective probes for monovalent cations. The tetrameric TPE derivative **35** shows a fluorescence enhancement in response to K^+^, forming a 1:2 **35**·K^+^ complex in which each cation is sandwiched between two crown ether units [112]. The BODIPY-based systems **36** and **37** respond to Cs^+^ [113].

Wang et al. have reported the tetra-crown ether/ TPE probe **35** which responds to K^+^ (Figure 15) [112]. Sensor **35** was synthesised by a thiol-ene reaction between a thiol-substituted TPE and a maleimide-substituted benzo-15-crown-5. This probe was found to be selective for K^+^ over different potentially interfering cations (Li^+^, Na^+^, NH_4_^+^, Ca^2+^, Mg^2+^ and Pb^2+^), with up to 9.5-fold fluorescence enhancement at 460 nm. The binding of K^+^ to the crown ether receptors of **35** was confirmed by a Job’s plot to involve formation of a 1:2 complex of K^+^ with the crown ether moieties. This leads to sandwich complexes of K^+^ with the different crown ethers, cross-linking the individual units of **35** in solution. Subsequent fluorescence quenching and restoration of the original emission spectrum of **35** upon addition of the unfunctionalised receptor, benzo-15-crown-5 to the **35**·K^+^ complex was observed; this supports an AIE mechanism, arising via the restriction of intramolecular rotation of the individual sensor units **35**. 

Depauw et al. have investigated the sensing of Cs^+^ using BODIPY-substituted calixarene-crown ether probes **36** and **37** (Figure 15) [113]. Upon the binding of Cs^+^ into the crown ether moiety of **36**, only very small increases to the fluorescence emission and quantum yield were observed, along with a red shift of the emission. In contrast, the less substituted BODIPY probe **37** gave a more pronounced fluorescence enhancement, red-shifted to 581 nm, and an increase in quantum yield. Theoretical calculations have attributed these differences to the position at which the BODIPY fluorophores are linked to the phenyl-crown ether receptor. In sensor **36**, the phenyl group is connected to the BODIPY via the *meso* position, and the aromatic systems are not coplanar with each other; this results in disrupted conjugation. In contrast, connection at the α-position as in **37** allows the phenyl-crown system to be conjugated with the BODIPY fluorophore.

**Figure 16:**
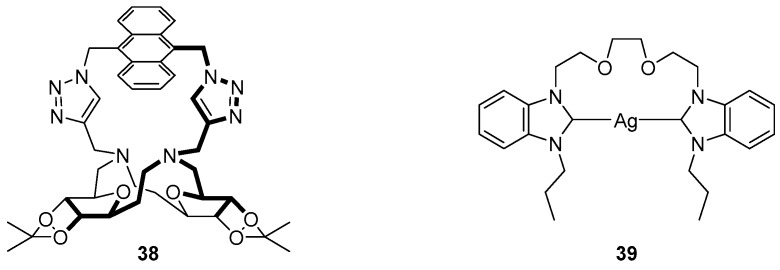
Crown ether anion sensors: anthracenyl derivative bis-triazole **38** reports HSO_4_^−^ binding with a significant increase in the anthracene emission [114], while *N*-heterocyclic carbene (NHC) complex **39** responds selectively to I^−^ with quenching of an emission band at 362 nm [116].

Crown ether-based sensors have also been used for the detection of anions. Yang et al. reported a triazole-linked anthracene substituted sugar-aza-crown ether sensor **38** for the detection of HSO_4_^−^ in methanol (Figure 16) [114]. The sensor **38** was highly selective for HSO_4_^−^, giving a significant fluorescence increase of the anthracene emission compared to the other competing anions tested (F^−^, Cl^−^, Br^−^, I^−^, NO_3_^−^, H_2_PO_4_^−^, AcO^−^) upon the formation of a 1:1 complex. ^1^H-NMR spectroscopic experiments demonstrated large downfield movements of the anthracene and triazole protons, suggesting coordination of HSO_4_^−^ to the triazole CH; this was confirmed by DFT calculations which revealed a lengthened triazole CH bond, supporting the proposal that the triazole CH functions as a hydrogen bond donor, an effect made possible due to the polarisation of the CH bond by the three triazole nitrogen atoms [115].

Liu et al. have reported a crown-based silver complex for sensing I^−^ [116]. The sensor **39** is an *N*-heterocyclic carbene (NHC) complex, with Ag^+^ coordinated to a *bis*-benzimidazolium system linked with an ether bridge. Fluorescence competition and titration experiments revealed the high selectivity of sensor **39** for the TBA^+^ salt of I^−^, with significant fluorescence quenching of the 362 nm peak upon the addition of up to 20 equivalents of I^−^, while no significant change to fluorescence emission was evident upon the addition of other anions (other halides, H_2_PO_4_^−^, HSO_4_^−^, AcO^−^ and NO_3_^−^) and counter-cations (K^+^, Na^+^, NH_4_^+^, Cu^+^ and Hg^2+^). Based on ^1^H-NMR, MS and FTIR experiments, I^−^ was proposed to coordinate to the Ag^+^ of **39** as a 1:1 complex leading to fluorescence quenching by an MLCT process.

## 7. Other Macrocyclic Sensors

**Figure 17:**
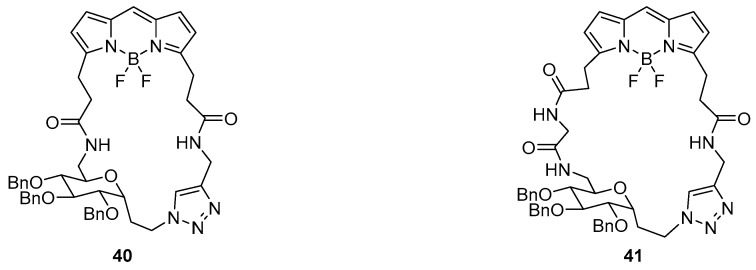
The sugar/ BODIPY conjugates **40** and **41** respond to both cations (Cu^2+^ and Fe^3+^) and anions (F^−^ and CN^−^), which quench the BODIPY emission at 515 nm. These anions are thought to strip BF_2_ from the BODIPY unit, while Cu^2+^ (triazole) and Fe^3+^ (carbonyls) coordinate to the macrocycle and quench emission by other means [117].

Sugar-based macrocycles containing a BODIPY fluorophore (**40** and **41**) have been reported as fluorescent sensors for both cations and anions (Figure 17), and were made utilising a CuAAC for the final macrocyclisation step [117]. Both probes demonstrated fluorescence quenching of the BODIPY emission peak at 515 nm in response to anions F^−^ and CN^−^ over a variety of other anions, and to Cu^2+^ and Fe^3+^ over other cations. The fluorescence quenching was investigated by ^11^B- and ^19^F-NMR spectroscopic experiments which revealed the disappearance of the BF_2_ signal upon the addition of F^−^ and CN^−^, suggesting quenching was due to the stripping of the BF_2_ moiety from BODIPY. Additional photophysical experiments with open-chain click precursors to sensors **40** and **41** revealed that triazole coordination is responsible for Cu^2+^ binding while the carbonyl groups were likely to be responsible for the coordination to Fe^3+^ [117].

**Figure 18:**
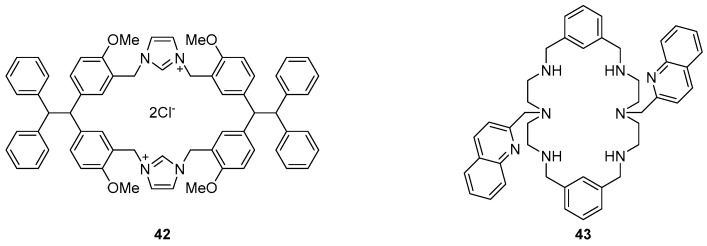
Combining Zn^2+^ with either of the macrocycles **42** and **43** affords highly effective probes for pyrophosphate anion (PPi). A solution containing TPE/imidazolium macrocycle **42** and one equivalent of Zn(OAc)_2_ reports PPi binding via an AIE mechanism, which gives rise to a significant fluorescence enhancement at 472 nm [118]. In a similar vein, the Zn^2+^ complex of methylquinoline-substituted azamacrocycle **43** has shown exceptional selectivity and sensitivity for PPi over other phosphorous-based anions, returning a 21-fold fluorescence increase in the presence of PPi (1 equivalent) via an ICT mechanism [119].

Two macrocyclic systems (Figure 18) have been developed as the basis of new selective sensors for pyrophosphate (PPi) anions. By itself, TPE-based imidazolium macrocycle **42** did not respond to any of the anions tested, including PPi, in a solution of 0.5% DMSO in water. However, the inclusion of one equivalent of Zn(OAc)_2_ in the solution led to a large fluorescence enhancement at 472 nm in the presence of PPi [118]. Competition experiments with other anions revealed no significant changes to emission. Other divalent cations (Cu^2+^, Ni^2+^, Pb^2+^, Co^2+^ and Cd^2+^) were tested but none approached the efficacy of Zn^2+^ at eliciting a fluorescence response to PPi. The size of the cavity in **42** is able to accommodate PPi as a 1:1 complex, a binding event that is driven by electrostatic attraction between the two positively charged imidazoliums and two of the negative charges on the pyrophosphate. The Zn^2+^ ion binds between the PPi components of up to four different **42**·PPi complexes to form aggregates of [**42**·PPi]_4_–Zn complexes which fluoresce by AIE.

Mesquita et al. recently reported PPi sensing with the zinc complex of methylquinoline-substituted azamacrocycle **43** (Figure 18) [119]. This system demonstrated exceptional selectivity and sensitivity for PPi over a variety of competing anions including other phosphorous-based anions, with a 21-fold fluorescence increase and small (10 nm) blue shift to 370 nm upon the addition of one equivalent of PPi. Binding takes place by coordination of two oxygens of the phosphoryl groups to the two Zn^2+^ ions bound inside the azamacrocycle, resulting in fluorescence enhancement due to changes to ICT in the quinolone ring.

**Figure 19:**
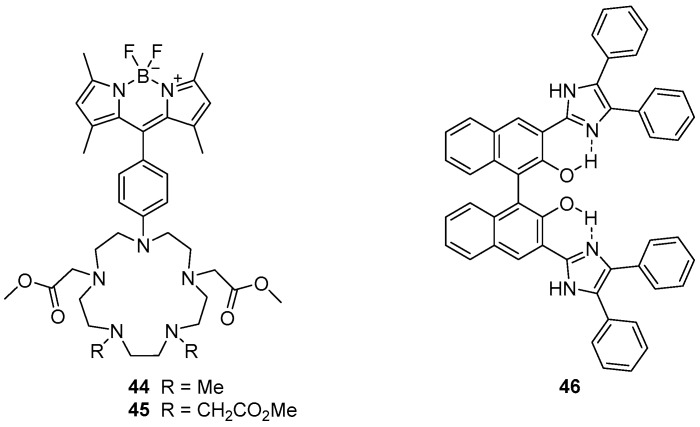
The unusual penta-azamacrocyclic receptor in **44** and **45** is selective for Mn^2+^, binding of which is reported by the appended BODIPY fluorophore via a mechanism thought to involve suppression of PET [120]. BINOL derivative **46** on the other hand is a ratiometric probe for Zn^2+^, binding of which can be monitored by following changes in fluorescence emission at 427 and 549 nm [121].

The penta-azamacrocycles **44** and **45**, which are appended with a BOIDPY fluorophore, been reported as ‘off-on’ sensors for Mn^2+^ (Figure 19) [120]. Selectivity for Mn^2+^ was observed, but some interactions with Zn^2+^ and Cu^2+^ were also evident. Binding of Mn^2+^ afforded 52- and 28-fold fluorescence enhancement of the BODIPY emission band at 508 nm for sensors **44** and **45** respectively which is significantly higher than the increase due to Zn^2+^ and Cu^2+^ binding. Mn^2+^ was shown to form 1:1 **44**·Mn^2+^ and 1:2 **45**·Mn^2+^ complexes, with Mn^2+^ binding inside the azamacrocycle and coordinating to two or four methyl esters. Consequently, these sensors fluoresce because PET from the azamacrocycle to the BODIPY unit is disrupted. The two sensors were applied to fluorescence confocal microscopy, and used to visualise Mn^2+^ localisation in doped HEK 293T cells.

## 8. Non-Macrocyclic Sensors

The diphenylimidazole substituted BINOL compound **46** has been reported as a sensor for Zn^2+^ (Figure 19) [121]. A variety of cations were tested and it was demonstrated that while Zn^2+^, Cu^2+^ and Hg^2+^ led to the quenching of the fluorescence emission at 549 nm, only Zn^2+^ gave rise to a new emission band at 427 nm. Fluorescence titrations with Zn^2+^ demonstrated the sensor **46** is a good ratiometric sensor, with linear relationships between the emissions at 427 and 549 nm and Zn^2+^ concentrations below 10 µM, via formation of a 1:1 complex. These ratiometric changes to the emission profile were proposed to result from inhibition of ESIPT upon Zn^2+^ binding to the phenol oxygen atoms and imidazole nitrogen atoms of **46**.

**Figure 20:**
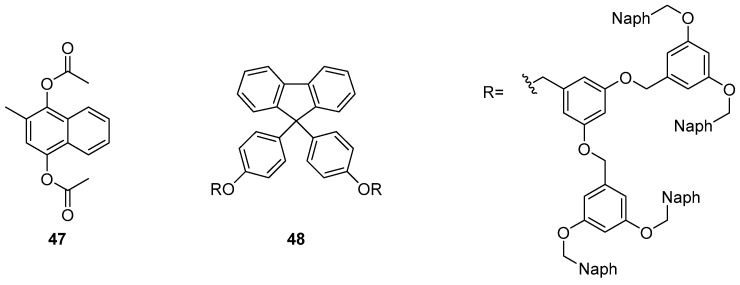
Vitamin K4 **47** is the substrate for dendrimeric probe **48**, which reports its presence with a 13-fold increase in the fluorescence emission at 340 nm. This is thought to arise via intermolecular energy transfer from the naphthalene branches of **48** to the aromatic target **47**, which is held within the naphthalene branches by π–π interactions. (Naph = 1-naphthalenyl) [122].

Adachi et al. reported the sensing of vitamin K4 **47** using a dendrimer-based sensor **48** which incorporates a fluorene fluorophore (Figure 20) [122]. A 13-fold fluorescence increase at 340 nm was observed in response to the addition of vitamin K4, along with a small fluorescence decrease in the excimer emission band at 440 nm. This fluorescence enhancement was rationalised by intermolecular energy transfer from the naphthalene branches of sensor **48** to target **47**, which is bound within the naphthalene branches of **48** by π–π interactions. This encapsulation of **47** also results in disruption of the original naphthalene-naphthalene stacked excimer emission of **48**.

**Figure 21:**
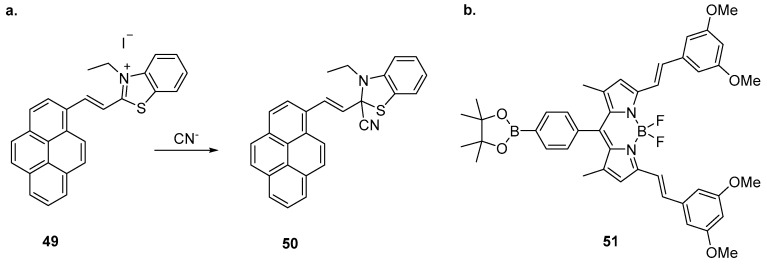
Non-macrocyclic fluorescent probes for anions. (**a**) Pyrene-based **49** is an effective sensor for CN^−^ anion, which reacts with the central carbon of the benzothiazolium component to form the emissive species **50** [123]; (**b**) BODIPY-functionalised boronate ester **51** responds to both CN^−^ and F^−^, even in the presence of various other competing anions. F^−^ triggers quenching and a blue shift in the emission of **51** from 599 nm to 579 nm, and new bands at 509 nm and 639 nm; CN^−^ also brings quenching and a blue shift to 575 nm, plus a single new emission at 478 nm [124].

Li et al. have reported the sensing of CN^−^ with a benzothiazole-based sensor incorporating a pyrene fluorophore (Figure 21) [123]. Sensor **49** was highly selective for CN^−^ over a variety of competing anions and demonstrated a significant fluorescence enhancement and blue shift from 604 nm to 470 nm, along with colorimetric changes to its absorption profile. The mechanism of action was investigated by ^1^H-NMR spectroscopy and MS, revealing that CN^−^ reacts with sensor **49** by nucleophilic addition to the benzothiazole C=N bond to form **50**. This results in the suppression of ICT leading to the ratiometric change in emission. Sensor **49** was effective between pH 5 and 9 and its application was successfully demonstrated using test strips of filter paper soaked with **49** which, upon exposure to CN^−^ solutions, produced colour changes detectable by eye and when visualised under 365 nm illumination.

The BODIPY-substituted boranate ester **51** has been reported to differentiate between CN^−^ and F^−^ anions (Figure 21) [124]. Significant colorimetric and fluorescence emission changes were seen upon the addition of CN^−^ or F^−^, changes unaffected by the presence of other species including competing anions (halides, AcO^−^, HSO_4_^−^, H_2_SO_4_^−^, NO_3_^−^, ClO_4_^−^). Fluorescence titrations revealed that F^−^ induced quenching and blue shift of the emission band of **51** from 599 nm to 579 nm, along with the appearance of two new emission bands centred at 509 nm and 639 nm. In contrast, CN^−^ induced quenching and blue shift to 575 nm while a single new emission band appeared at 478 nm. The difference in photophysical response was proposed to arise from differences between the boron Lewis acidic binding sites on **51**. ^1^H-NMR spectroscopic experiments suggested that CN^−^ binds at the BODIPY moiety, leading to the displacement of BF_2_ while F^−^ binds at both the BF_2_ and the pinacol boron atom to generate an *sp*^3^ hybridised boronate ion.

**Figure 22:**
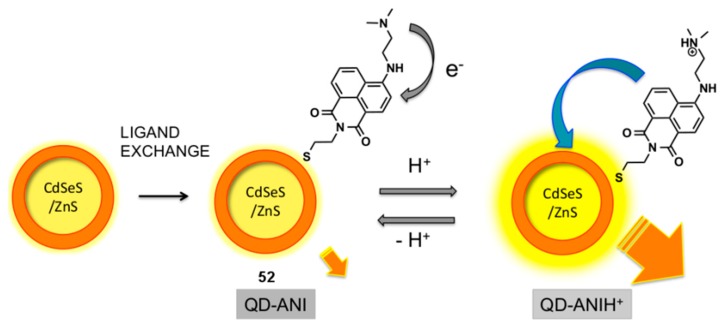
Quantum dot (QD)/aminonaphthalimide (ANI) conjugate that functions as a pH-responsive probe. Reproduced from reference [125], Published by the PCCP Owner Societies under a Creative Commons Attribution 3.0 Unported Licence (CC BY 3.0).

Quantum dot (QD)/aminonaphthalimide (ANI) conjugates have been studied by Ast et al. and evaluated as pH probes (Figure 22) [125,126]. The QD-ANI conjugate **52** was assembled by a mass-driven ligand exchange process which attached the ANI dyes onto the surface of the QD via the displacement of capping ligands on the QD, with the naphthalimide-thiols generated in situ from their disulfide dimers. It was found that the fluorescence output of the Cd-SeS-ZnS QD at 571 nm was enhanced by the attachment of ANI fluorophores, via FRET from the dye to the dot. The QD-ANI **52** functioned as a pH probe, with a sigmoidal relationship between its integrated emission intensity and pH, and increasing fluorescence intensity at lower pH. The increase in fluorescence intensity at lower pH was rationalised by the interruption of PET from the dimethylamino group into the naphthalimide in ANI due to protonation, which modulates FRET from the dye to the QD.

## 9. Conclusions and Outlook

Over the past few years, the already large and diverse collection of synthetic fluorescent sensors has continued to expand rapidly, with many new probes developed by combining a range of binding moieties with various fluorescent reporters. These sensors operate via a variety binding modes and different mechanisms of fluorescence response, allowing the selective detection of numerous analytes from cations and anions to small molecules and biomolecules. However, many challenges remain to improve substrate scope and selectivity, tolerance to pH, temperature and solvent, and other subtle nuances of probe behaviour to enable their broad application in complex, real-world systems.

Of the more than 60 sensor systems discussed in this review, 60% are probes for cations, 27% respond to anions, and the remaining 13% signal the presence of other species. There is a relative abundance of probes for Zn^2+^, Cu^2+^ and Hg^2+^, with 21 (35%) of the systems discussed responding to one or more of these three metal ions; in comparison, we have discussed two probes each for Cr^3+^, Mn^2+^ and Fe^3+^, and one for each of H^+^, K^+^, Ca^2+^, Co^2+^, Ag^+^, Cd^2+^, Cs^+^ and Pb^2+^. Amongst the anion probes four respond to CN^−^, three to F^−^, two each to HSO_4_^−^, S^2−^ and PPi, and one each to HS^−^, H_2_PO_4_^−^ and I^−^. The probes for ‘other species’ comprise three for small molecules (nitroxyl (2) and formaldehyde) and one for each of the larger entities biotin, spermine, buckminsterfullerene, TNT and vitamin K4. Clearly there is an uneven distribution of capacity here, and a great many important potential substrates—both biomedically and environmentally relevant species—are not represented at all. This unevenness reflects in part the relative difficulty of designing receptors that specifically bind neutral molecules or anions compared to systems that bind positively charged species, versus the relative abundance of readily accessible macrocyclic receptors with high affinity for first-row transition metal cations. However, this situation also arises in part from a tendency towards synthesis-driven ‘retrofitting’ of probes to targets, whereby probes are made using established synthetic methods that allow the modular combination of receptor and transducer components, then the optimal substrate determined via a screening process. The alternative ‘bottom up’ design and synthesis of probes for specific substrates is more challenging—particularly when it comes to substrates for which high affinity binding motifs must first be established—but ultimately more sensors of this ilk are required to expand the range of substrates that can be monitored with fluorescent probes.

The wider uptake and application of these systems into general usage is another key unmet challenge. As recently discussed in more detail elsewhere, very few of the fluorescent probes reported in the literature ever find broad application [5]. Indeed the majority are never discussed again beyond the initial publication reporting their synthesis and evaluation. In addition to expanding the range of substrates that can be followed, meeting the challenge of genuine utility in biological contexts requires a greater focus on probes that emit in the infrared or near-infrared region of the spectrum and are thus ‘visible’ in vivo [127], plus greater emphasis on ratiometric systems (vide supra), which mitigate against variations in probe concentration that are difficult to avoid in complex biological systems. Greater attention to solubility in aqueous systems is also necessary, towards development of a broad suite of water-compatible probes; while some of the systems detailed in this review do indeed function under genuinely aqueous and physiologically relevant conditions, others use ‘aqueous conditions’ that in fact contain as little as 20% *v*/*v* H_2_O. Lastly, a broader and more subtle array of methods for targeting probes to specific cell types, extracellular and subcellular locations is also needed to extend the usefulness of these systems in biological experiments [5,128,129].

Other exciting developments are unfolding apace, including the use of multiple fluorescent reporters in sensing arrays, and combining small-molecule chemosensors with smartphone detectors as portable spectrophotometers for remote measurement. By using an array of sensors and then analysing their fluorescence responses statistically, it becomes possible to identify distinctive response patterns for each analyte and thus circumvent selectivity limitations of the individual chemosensors when used in isolation [130]. Combining this approach with the development of multi-channel sensors capable of detecting more than one analyte at a time [131], highly robust sensor arrays can be envisaged.

Meanwhile rapid developments in smartphone technologies, particularly improvements in processing power and the quality of camera CMOS sensors, are enabling the application of these ubiquitous devices as highly portable spectrometers for real-time fluorescence-based monitoring and analysis of analytes on-site [132,133,134]. While a performance gap remains between smartphone systems and laboratory spectrometers with regards to sensitivity and detection limits, continued hardware improvements, coupled with advances in 3D printing and other enabling technologies, will alleviate this. Smartphone based systems offer real promise for affordable, portable fluorescence-based sensing applications in the near future.
